# Biological horizons: pioneering open science in the cosmos

**DOI:** 10.1038/s41467-024-48633-2

**Published:** 2024-06-11

**Authors:** Sylvain V. Costes, Chelle L. Gentemann, Steven H. Platts, Lisa A. Carnell

**Affiliations:** 1 Space Biosciences Research Branch, NASA Ames Research Center, Moffett Field, CA USA; 2 Office of the Chief Science Data Officer, Science Mission Directorate, NASA Headquarters, Washington DC on Intergovernmental Personnel Act assignment from the International Computer Science Institute, Berkeley, CA USA; 3 Human Research Program, NASA Johnson Space Center, Houston, TX USA; 4 Biological and Physical Sciences Division, NASA Headquarters, Washington, DC USA

**Keywords:** Space physics, Cell biology, Research data

With precious few spaceflight missions, all space-related biological and biomedical-health data are priceless national resources that anyone should be able to easily access. The NASA Open Science Data Repository (OSDR: https://osdr.nasa.gov/bio) plays a crucial role in ensuring such accessibility, housing comprehensive space-related datasets derived not only from various model organisms but also from non-NASA human astronauts^1^. These datasets cover a wide spectrum of biological assays conducted at multiple levels, ranging across cells, tissues, organs, and whole organisms. They effectively capture diverse types of data, including ‘omics, phenotypic, physiological, and behavioral changes experienced in space^2,3^. Additionally, OSDR enriches this data by providing metadata contextualizing each experiment including the associated experimental hardware and environmental telemetry. OSDR has worked with a large and open scientific community to standardize data generation and formatting, enabling advanced analytics, including the use of artificial intelligence (AI) for data analysis.

With the support of NASA’s scientific leadership, the spirit of Open Science has permeated every scientific division at NASA, including the Biological and Physical Science (BPS) Division. The BPS Division has emerged as a pivotal force in fueling humanity’s fascination with space exploration by understanding the mechanisms of life adapting to extreme environmental hazards of deep space^4^. As NASA launched the ambitious "Transform to Open Science" program (TOPS)^5^ in 2023, it aims to accelerate the adoption of a more reproducible and transparent approach to research. TOPS also aims to train 20,000 scientists in the art of Open Science by 2028 (https://nasa.github.io/Transform-to-Open-Science/), pushing for better equity and inclusivity in science. Within the BPS Division, the curation of an extensive array of space biological, space health, and mission telemetry data in standardized formats has been vetted by over 500 citizen scientists, members of the NASA OSDR Analysis Working Groups (AWG - https://osdr.nasa.gov/bio/awg/join.html). As a result, OSDR system ensures the invaluable data’s findability, accessibility, interoperability, and reusability (FAIR)^6^, despite the challenges posed by its multi-hierarchical, multimodal, and heterogeneous nature.

As a celebration of 2023 being the year of Open Science at NASA, a Nature portfolio Collection titled "The Second Space Age: Omics, Platforms, and Medicine Across Orbits^7^,’’ is a series of inspiring articles enabled by OSDR, enriched by new data generously shared by civilian astronauts aboard the SpaceX Inspiration4 mission. The investigator team involved in this work has embraced Open Science by sharing research data and enabling data mining by the AWG community. Data presented in this Nature portfolio Collection were fully utilized because of the open and accessible nature of the OSDR system. This array of work shows how NASA handled the storage and reuse of a diverse range of data from molecular to ecosystem levels, encompassing tabular, omics, imaging, code, video, biospecimens, and environmental physical-chemical telemetry data. The AWGs played a critical role standardizing metadata and data processing pipelines in OSDR, enabling groundbreaking discoveries in space biology and biomedical health by making meta-analysis possible. In this context, two recent articles involving the AWGs were published in Nature Machine Intelligence and featured in a special issue on AI, detailing the future role of AI and Open Science in deep space biological research^8^ and biomedical health support^9^, describing how Open Science and standardization integrated with AI can maximize the value of limited yet vital spaceflight data.

The spirit of Open Science and the invaluable insights from space biology have transcended boundaries, extending their embrace to other vital groups within NASA, including the Human Research Program (HRP) focused on the health and performance of NASA Astronauts. This Open Science approach champions practical, collaborative scientific discovery, with an emphasis on data sharing and immediate applications. Insights from space biology, facilitated by Open Science, are vital not just for aspirational goals like moon and Mars missions, but for direct advancements in human health and technology on earth. This Nature portfolio Collection highlights how collective efforts and open data can drive substantial scientific progress, merging the thrill of cosmic exploration with tangible and immediate benefits.
